# Determination of the Precision of Glucometers Used in Saudi Arabia

**DOI:** 10.3390/s25113561

**Published:** 2025-06-05

**Authors:** Shoug A. Al-Othman, Zahra H. Al-Zaidany, Shahad H. Al-Ghannam, Ahmed M. Al-Turki, Abdulrahman A. Al-Abdulazeem, Chittibabu Vatte, Alawi Habara, Amein K. Al-Ali, Mohammed F. Al-Awami

**Affiliations:** 1 College of Medicine, Imam Abdulrahman Bin Faisal University, Dammam 31441, Eastern Province, Saudi Arabia; 2210000929@iau.edu.sa (S.A.A.-O.); 2210000985@iau.edu.sa (Z.H.A.-Z.); 2210000604@iau.edu.sa (S.H.A.-G.); 2210000289@iau.edu.sa (A.M.A.-T.); 2210000708@iau.edu.sa (A.A.A.-A.); 2 Department of Biochemistry, College of Medicine, Imam Abdulrahman Bin Faisal University, Dammam 31441, Eastern Province, Saudi Arabia; cbvatte@iau.edu.sa (C.V.); aalali@iau.edu.sa (A.K.A.-A.); 3 Cavendish Laboratory, University of Cambridge, JJ Thomson Avenue, Cambridge CB3 0HE, UK; mfaa2@cam.ac.uk; 4 Cambridge Nucleomics, 254 Turning Way, Cambridge CB3 1AF, UK

**Keywords:** glucometer, precision, specificity, vitamin C, acetaminophen, maltose

## Abstract

**Highlights:**

**What are the main findings?**

- The prevalence of self-monitoring blood glucose among individuals diagnosed with diabetes in Saudi Arabia is 95%, using commercially available glucometers.
- Among the three glucometers tested, only Accu-Chek Instant met precision standards, while ConTour showed significant positive interference from vitamin C, exceeding acceptable bias limits.

**What is the implication of the main finding?**

- Regular use of glucometers is widespread, underscoring the importance of evaluating device accuracy and reliability.
- Certain glucometers may produce misleading glucose readings in the presence of interfering substances like vitamin C, highlighting the need for greater awareness among healthcare providers and patients.

**Abstract:**

Background: Efforts have been joined to set the parameters for the reliability of glucometers, yet once they are on the market, they are not further tested for the maintenance of accuracy, specificity, or precision. Methods: This comparative analytical study investigated the precision of commonly used glucometers in Saudi Arabia, namely Accu-Chek Instant^®^, On-Call Sharp^®^, and ConTour^®^, as well as the effects of vitamin C, acetaminophen, and maltose on glucose readings. Ten milliliters of blood was drawn in lithium heparin from healthy volunteers (n = 9). Six samples were divided into two groups of three. One group was designed for normal glucose levels. The second group was designed for high glucose levels by adding a dextrose solution. The last three samples were designed for low glucose levels by leaving the sample for 24 h at room temperature and then following with centrifuge and plasma extraction. Results: This study showed that only Accu-Chek Instant met the International Organization for Standardization (ISO) standard for precision across all dextrose concentrations, along with intra-class correlation values ranging from 0.95–1 (*p* < 0.001). By spiking the plasma samples with sub-therapeutic, therapeutic, and overdose concentrations of the metabolites, we found that vitamin C had a more evident interference on glucose readings compared to acetaminophen and maltose. Conclusions: The ascertainment of the precision of glucometers and the effects of interferences on them are vital in preventing the improper administration of insulin, which can lead to serious complications.

## 1. Introduction

Diabetes mellitus (DM) is a set of chronic, metabolic disorders that are characterized by hyperglycemia, oxidative stress, and inflammation [1]. In type I diabetes, the pancreas is unable to produce sufficient insulin for glucose uptake due to an autoimmune response against beta-cells. On the other hand, type II diabetes is involved in progressive insulin resistance in the cells of the body and the eventual failure of the pancreas [1,2]. According to the World Health Organization, Saudi Arabia has the second-highest rate of diabetes in the Middle East and is ranked seventh globally [3].

Glucometers are electrochemical biosensors which have dehydrated and immobilized bioreceptors, such as glucose oxidase or glucose dehydrogenase, inserted in one of two or three electrodes used on the test strip for the detection of a stimulus with a transduction element that converts current to voltage [4,5,6,7]. Finally, a signal processing device detects and converts the voltage into digital signals for the liquid crystal display (LCD) panel [4].

The glucose biosensing mechanism depends on a redox reaction (Supplementary Figure S1). In the reductive half, β-D-glucose becomes oxidized to glucono-δ-lactone by an enzyme (e.g., glucose dehydrogenase), while its cofactor, such as flavin adenine dinucleotide (FAD), is reduced [8,9]. It has been reported that commonly used medications, such as Vitamin C, maltose, and acetaminophen, influence the readings provided by various glucometers [10,11].

Medications and supplements used in the treatment of diabetes can interfere with glucometer readings. First, vitamin C is given to lower blood glucose levels and treat free-radical damage or oxidative stress in diabetic patients, which means that it can affect the oxidative half of the redox reaction [1]. There is no consensus in the scientific literature regarding the effect of vitamin C on glucometer measurements, as some studies show conflicting results based on the device used [10]. Secondly, acetaminophen is widely used for mild pain, even among diabetic patients [12]. This analgesic also affects the oxidative half by undergoing oxidation at its phenol end when exposed to the sensory electrodes of a glucometer, giving off electrochemical signals other than that of blood glucose. Hence, the readings projected by the device can vary depending on the device used [13,14]. Lastly, maltose is a glucose–glucose disaccharide and is incorporated in prophylaxis and IV chemotherapeutic treatments as a regulatory agent. Glucometers detect the sum glucose concentration in a solution, including the bound glucose molecules found in injected maltose; therefore, the overall elevated concentration can be easily misinterpreted as elevated serum glucose levels [15]. Understanding the effects of major interferences on the glucose monitoring system is vital in preventing excess or scarce administration of insulin, which can lead to fatal complications such as hypoglycemia-induced death [16].

According to the current literature, the precision and specificity for three commonly used glucometers in Saudi Arabia, namely, Accu-Chek Instant and On-Call Sharp, have not been tested before, while ConTour was tested to confirm the results of previous studies. Studies on glucometers in Saudi Arabia are scarce, and there is a need to conduct more research in this respect and specifically on the precisions of these glucometers [17]. Therefore, the aims of this investigate are to test the precision of three commonly used glucometers in Saudi Arabia, namely, Accu-Chek^®^ Instant, On-Call^®^ Sharp, and ConTour^®^, using plasma-spiked samples of different glycemic ranges (low, normal, and high), and how vitamin C, acetaminophen, and maltose affect the glucose readings.

## 2. Materials and Methods

**Figure 1:**
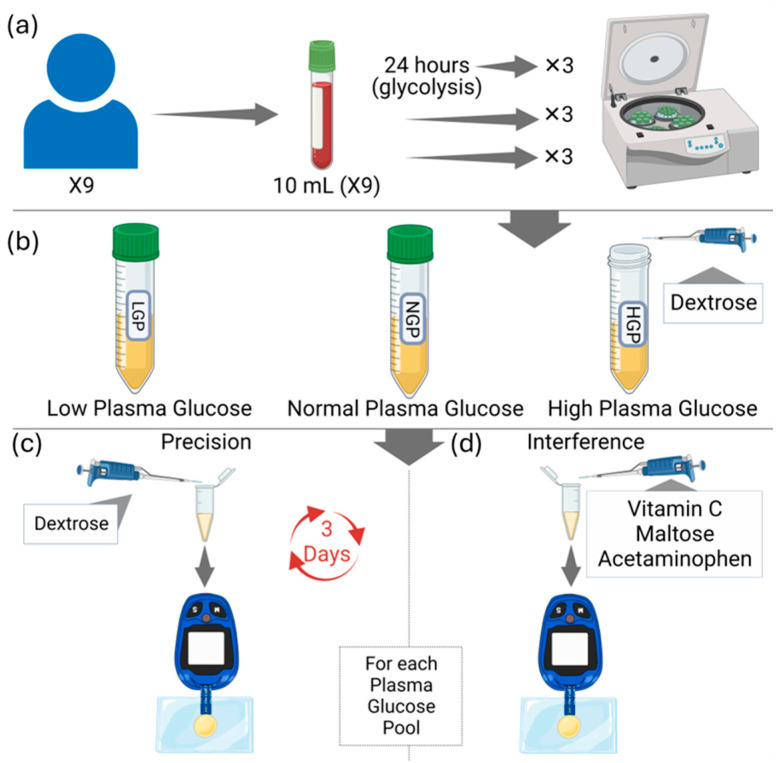
Experimental procedures of this study. (**a**) Ten mL of blood was drawn from nine healthy donors. The donors were divided into three equal groups (three subjects each), the plasma was extracted from two groups immediately after blood extraction, and each group was pooled together. The blood samples from the third group were left for 24 h at room temperature prior to plasma extraction and pooling of the third group (low glucose level). (**b**) Up to 400 mg/dL of dextrose was added to one of the first two groups to make a high glucose level, while the second of the first two groups represented the normal blood glucose level. (**c**) For the precision experiment, different concentrations of dextrose were added to a sample of each plasma pool to make five aliquots for each pool. Then, the aliquots were stored at 4 °C, and then these aliquots were used for the precision experiments on days 2 and 3. (**d**) For the interference experiment, the steps in section (**c**) were performed, but potentially interfering substances were added to six aliquots and only for one day.

Volunteers from the student body of the College of Medicine, Imam Abdulrahman bin Faisal University (n = 9) were requested to participate in this study. A written informed consent was signed by each participant. Ethical approval of this study was obtained from the Imam Abdulrahman bin Faisal University Institutional Review Board Committee (IRB# 2019-01-112) and this study was conducted according to the ethical principles of the Declaration of Helsinki and Good Clinical Practice guidelines. All participants were examined by a physician at the King Fahd Hospital of the University to ensure that these volunteers were healthy. The precision of three commonly used glucometers in Saudi Arabia, namely, Accu-Chek Instant^®^ (Roche Diabetes Care, Indianapolis, IN, USA) On-Call Sharp^®^ (CON Laboratories, Inc., San Diego, CA, USA), and ConTour^®^ (Ascensia Diabetes Care, Basel, Switzerland)—were tested using normal plasma and maltose-spiked plasma samples. Ten milliliters of blood was drawn in lithium heparin coated vacutubes by a qualified phlebotomist. The blood samples (n = 6) were centrifuged at 3500 RPM for 5 min, and the plasma were extracted into new, clean tubes (Figure 1).

To prepare normal glucose level plasma pools of different glycemic ranges, three of the six plasma samples were combined and mixed by vortexing. The remaining three plasma samples were combined, and from the total volume of 15 mL, 4 mL was spiked with 0.04 mL of a 400 mg/mL dextrose solution. This addition resulted in a final glucose concentration of 400 mg/dL, creating the high blood glucose pool. These two pools were stored at 4 °C until used. To prepare the low blood glucose pool, the remaining three whole blood tubes were left to stand for 24 h at room temperature, followed by centrifugation and extraction of the plasma. Hence, a total of three pools were obtained (high, normal, and low). The exact glucose levels of these three pools were determined using glucose (HK) assay kit (Sigma Aldrich, St. Louis, MO, USA) (Supplementary Table S1). From the baseline aliquots of each plasma pool, multiple 10 µL drops of plasma were placed on a parafilm, and five glucose readings were taken by each glucometer for 3 days for a total of 135 values. The baseline results were considered as the control group for the interference experiment. A highly concentrated stock solution was prepared by adding 3 mL of water to 1200 mg, 600 mg, 2400 mg, and 300 mg of dextrose, vitamin C, maltose, and acetaminophen, respectively. Diluted solutions were obtained using the serial dilution method determined by the equation V1 × M1 = V2 × M2, where V1 is the total spiked plasma volume, M1 is the final desired plasma concentration, V2 is the volume of diluted solution added to the plasma, and M2 is the concentration of the diluted solution. The aliquots were mixed using a vortex and then stored at 4 °C. In regard to preparing the acetaminophen, the acetaminophen was dissolved in ethanol as acetaminophen is sparingly soluble in water. According to the International Federation of Clinical Chemistry and Laboratory, the volume of the diluting solution added to the plasma sample should be less than 10% of the final volume of both mixes. In this study, the volume of the solution added to the plasma equals 1% of the total volume of plasma and solution (V1) mixed to minimize the potential error resulting from dilution; accordingly, the volume of solution added to a 198 µL plasma sample was 2 µL.

The baseline aliquot of each pool (41, 97, and 413 mg/dL pools, which represent low, normal, and high glycemic levels, respectively) was subsequently divided into five micro-tubes for the precision experiment. Each micro-tube was spiked with a different dextrose concentration (20, 40, 60, 120, and 400 mg/dL). Five readings were taken by each glucometer device per micro-tube from each plasma pool. Following this, the aliquots were stored at 4 °C, and the same steps were repeated for two more consecutive days (days 2 and 3). A total of 675 glucose readings were recorded. The experimental work was conducted on samples that were brought to room temperature.

The remaining baseline plasma samples of each blood pool were divided into six aliquots per metabolite and spiked with different concentrations of interfering substances. Ultimately, three readings were taken by each glucometer for each metabolite concentration. The total readings obtained were 486 glucose readings. For vitamin C, 5, 10, 25, 50, 100, and 200 mg/dL were added to plasma samples whose glucose levels had been adjusted to approximately 41, 97, and 413 mg/dL. For maltose, 10, 40, 200, 480, 600, and 800 mg/dL were added to plasma samples whose glucose levels had been adjusted to 41, 97, and 413 mg/dL. And finally for acetaminophen, 0.1, 5, 20, 50, 80, and 100 mg/dL were added to plasma samples whose glucose levels had been adjusted to 41, 97, and 413 mg/dL.

The data obtained from the experiment were analyzed using the Social Package of Statistical Science (SPSS), version 28.0.1.1, and the statistical significance was set at *p* < 0.05. For the precision experiments, the results were expressed as the percentage of the coefficient of variation of the glucose readings using the following formula: (standard deviation/mean) × 100, assessing both the within-run and between-run precision. The glucose readings used to calculate the coefficient of variation percentage (%CV) for the within-run precision were only those of the first day, while those of the between-run precision included the readings across the 3 days. For interference experiments, results were expressed as mean change from baseline (glucometer with interfering substance—glucometer at baseline) in mg/dL for the samples, with the glucose concentration adjusted to <100 mg/dL. In samples with glucose levels adjusted to >100 mg/dL, the mean change from baseline in percent [(glucometer with interfering substance − glucometer at baseline)/(glucometer at baseline) × 100] was used. The results were assessed according to the 2013 ISO 15197 standards [18], which state that a %CV should be ≤ 5% at glucose concentrations ≥ 100 mg/dL. The intraclass correlation (ICC) was also used for the precision experiment. The average measures calculated by SPSS were interpreted based on the following cutoff points, along with their confidence intervals for statistical significance [19]:

- 0–0.5 = poor reliability;
- 0.5–0.75 = moderate reliability;
- 0.75–0.9 = good reliability;
- 0.9–1 = excellent reliability.

For the interference experiment, the mean difference, or bias, between the test samples and the control samples did not exceed 10 mg/dL at glucose concentrations < 100 mg/dL or within bias percentages of ±10% at glucose concentrations ≥ 100 mg/dL. For key resources, see Supplementary Table S4.

## 3. Results

### 3.1. Baseline

At the low blood glucose level, the means of the glucose readings (mg/dL) were 31.3 ±1.2 for Accu-Chek Instant, 26.1 ± 3.1 for On-Call Sharp, and 37.7 ± 1.1 for ConTour (Supplementary Figure S2a). At the normal blood glucose level, the means of the glucose readings (mg/dL) were 110.8 ± 3.3 for Accu-Chek Instant, 115.2 ± 3.8 for On-Call Sharp, and 114.5 ± 7.9 for ConTour (Supplementary Figure S2b). At the high blood glucose level, the means of the glucose readings (mg/dL) were 375.6 ± 10.4 for Accu-Chek Instant, 387.9 ± 11.2 for On-Call Sharp, and 393.5 ± 12.7 for ConTour (Supplementary Figure S2c).

### 3.2. %CV

**Figure 2:**
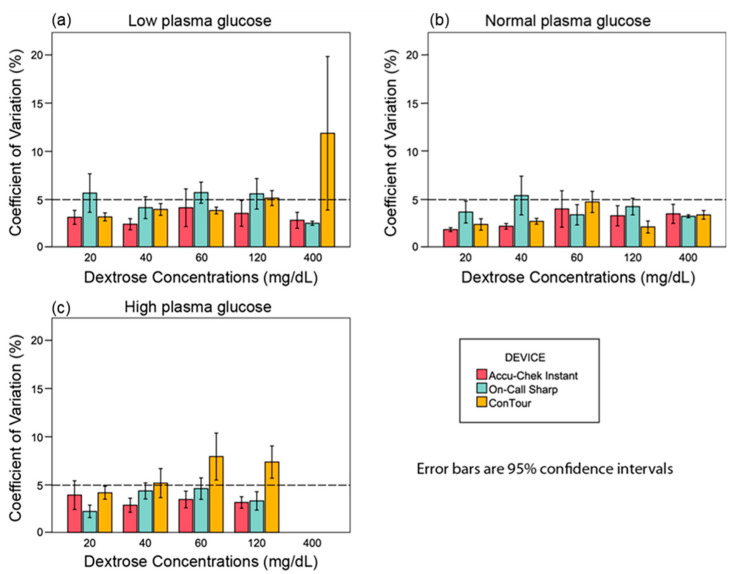
Coefficient of variation percentage (%CV) versus dextrose concentration bar charts for the between-run precision experiment. (**a**) The %CV at different dextrose concentrations in the low plasma pool. (**b**) The %CV values at different dextrose concentrations in the normal plasma pool. (**c**) The %CV at different dextrose concentrations in the high plasma pool. The %CV of the glucose readings equals the [(standard deviation/mean) × 100]. Missing values in the high plasma pool are due to error messages.

In the samples adjusted to 41 mg/dL of glucose, Accu-Chek Instant constantly yielded %CV values that were within the normal limits, ranging from 2.3% to 4%. On-Call Sharp continuously gave %CVs above 5%, ranging from 5.5% to 5.6% except when spiked with 40 and 400 mg/dL of dextrose that yielded 4% and 2%, respectively. On the other hand, the %CV of ConTour was constantly below 5%, ranging from 3.1% to 3.9% except at 120 and 400 mg/dL of dextrose, which yielded 5.1% and 11.8%, respectively (Figure 2a).

For the plasma samples adjusted to 97 mg/dL of glucose, all devices continuously gave %CV values below 5% except for On-Call Sharp, which, when spiked with 40 mg/dL of dextrose, yielded 5.3%. The %CV values ranged from 1.8% to 3.9% for Accu-Chek Instant, 3.2% to 4.1% for On-Call Sharp, and 2.1% to 4.7% for ConTour (Figure 2b).

In the samples adjusted to 413 mg/dL of glucose, Accu-Chek Instant and On-Call Sharp constantly gave %CV values below 5% that ranged from 2.8% to 3.9% and 2.2% to 4.5%, respectively. On the other hand, ConTour yielded %CV values above 5%, ranging from 5.1% to 7.9%, except at 20 mg/dL of dextrose, which gave 4.1% (Figure 2c). At 400 mg/dL of dextrose, all meters yielded error messages due to the extensively high glucose concentration.

Further analysis can be found in Supplementary Tables S5 and S6 in the Supplemental Information.

### 3.3. Intraclass Correlation (ICC)

For the samples adjusted to 41 mg/dL of glucose, all three devices gave statistically significant, excellent intra-rater reliabilities: both Accu-Chek Instant and On-Call Sharp (ICC: 0.999; 95% CI: 0.998 to 1; *p* < 0.001) and ConTour (ICC: 0.997; 95% CI: 0.990 to 1; *p* < 0.001).

For the samples adjusted to 97 mg/dL of glucose, all three devices also had a statistically significant, excellent intra-rater reliability: Accu-Chek Instant (ICC: 1; 95% CI: 0.999 to 1; *p* < 0.001), On-Call Sharp (ICC of 0.999; 95% CI: 0.996 to 1; *p* < 0.001), and ConTour (ICC: 0.998; 95% CI: 0.994 to 1; *p* < 0.001).

For the samples adjusted to 413 mg/dL of glucose, Accu-Chek Instant had a statistically significant, excellent intra-rater reliability (ICC of 0.945; 95% CI: 0.818 to 0.996; *p* < 0.001), while On-Call Sharp gave a statistically significant, good intra-rater reliability (ICC: 0.897 (95% CI: 0.618 to 0.997; *p* < 0.001), and ConTour gave a moderate intra-rater reliability (ICC: 0.688; 95% CI: −0.111 to 0.992; *p* < 0.042).

### 3.4. Interference Data

#### 3.4.1. Vitamin C

**Figure 3:**
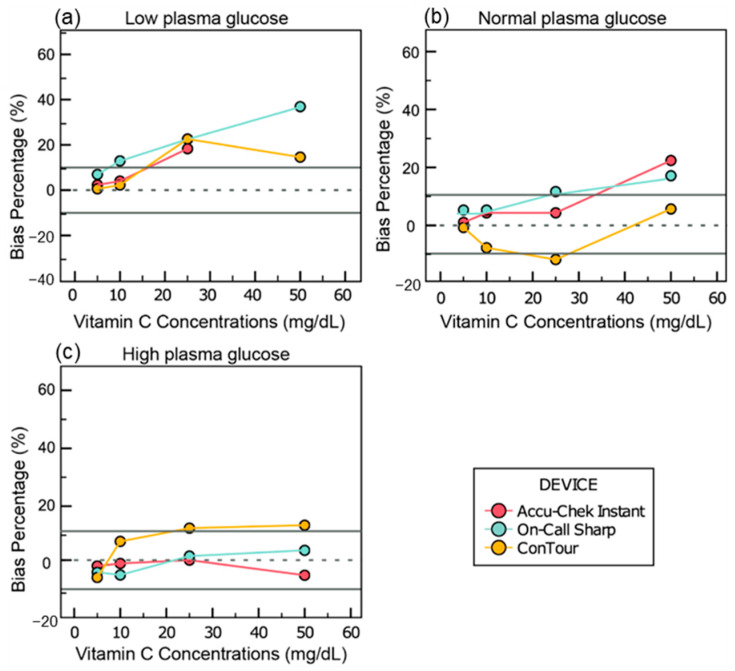
Bias and bias percentages of glucose readings across various vitamin C concentrations. The X-axes of all graphs show vitamin C levels. The Y-axes represent (**a**) the bias calculated as [glucometer reading in maltose-spiked samples (mg/dL) − baseline reading (mg/dL)] for samples adjusted to 41.16 mg/dL of glucose and (**b**,**c**) the bias percentage calculated as [(glucometer with maltose − baseline glucometer)/(baseline glucometer) × 100] for samples adjusted to 96.9 mg/dL and 413.26 mg/dL of glucose, respectively. The missing value for the Accu-Chek Instant at the low plasma pool is due to error messages.

In the samples adjusted to 41 mg/dL of glucose, concentrations of 25 mg/dL or higher of vitamin C changed the glucose readings by 18.3 mg/dL of glucose for Accu-Chek Instant, 22.6 to 114.6 mg/dL for On-Call Sharp, and 14.6 to 22.6 mg/dL for ConTour (Figure 3a).

For the plasma samples adjusted to 97 mg/dL of glucose, bias percentages outside the range of −10% to 10% were yielded at vitamin C concentrations of 50 mg/dL for Accu-Chek Instant (21.8%), ≥25 mg/dL for On-Call Sharp (11.1% to 64.4%), and 25 mg/dL for ConTour (−12.4%) (Figure 3b).

In the samples adjusted to 413 mg/dL of glucose, changes of 11.1% and 12.1% in the glucose readings were observed in ConTour when 25 and 50 mg/dL of vitamin C were added to the samples, respectively, and 12.2% with Accu-Chek Instant when 100 mg/dL of vitamin C was added (Figure 3c).

In 41 mg/dL of glucose, when 50 mg/dL of vitamin C was added, Accu-Chek Instant yielded E12 error messages. Moreover, when 100 and 200 mg/dL of vitamin C were added, Accu-Chek Instant and ConTour yielded E3 and E11 error messages, respectively. In 97 mg/dL glucose, when 100 and 200 mg/dL of vitamin C were added, Accu-Chek Instant and ConTour yielded E12 and E11 error messages, respectively. In 413 mg/dL of glucose, when 100 mg/dL of vitamin C was added, ConTour yielded E11 error messages, while with 200 mg/dL of vitamin C, Accu-Chek Instant and ConTour displayed E3 and E11 error messages, respectively.

Further analysis can be found in Table S7 along with the graphs showing all vitamin C concentrations (Figure S3) in the Supplemental Information.

#### 3.4.2. Maltose

**Figure 4:**
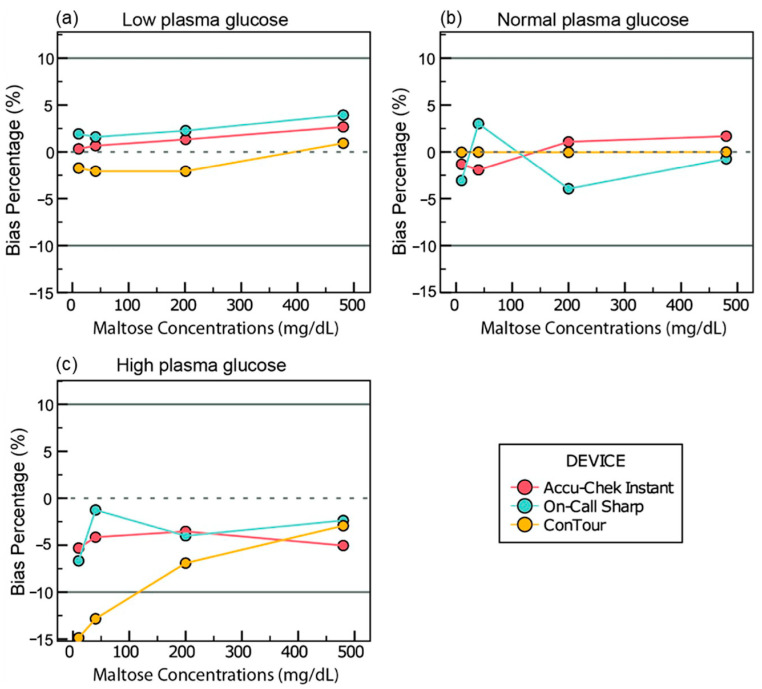
Bias and bias percentages of glucose readings across various maltose concentrations. The X-axes of all graphs show maltose levels. The Y-axes represent (**a**) the bias calculated as [glucometer reading in maltose-spiked samples (mg/dL) − baseline reading (mg/dL)] for samples adjusted to 41.16 mg/dL of glucose and (**b**,**c**) the bias percentage calculated as [(glucometer with maltose − baseline glucometer)/(baseline glucometer) × 100] for samples adjusted to 96.9 mg/dL and 413.26 mg/dL of glucose, respectively.

In the samples adjusted to 41 and 97 mg/dL of glucose, all meters displayed a change between −10 and 10 mg/dL of glucose (Figure 4a,b). The change in the samples of 41 mg/dL of glucose ranged from 0.3 to 3.3 mg/dL of glucose for Accu-Chek Instant, 1.6 to 6.9 mg/dL for On-Call Sharp, and −2 to 0.9 mg/dL for ConTour. Meanwhile, Accu-Chek showed a change from −1.9% to 2.9% in the samples adjusted to 97 mg/dL at different maltose concentrations, while On-Call Sharp yielded changes ranging from −3.9% to 3% and ConTour yielded changes from −0.04% to 0.02%.

In the samples adjusted to 413 mg/dL of glucose, ConTour showed a change of −14.9% and −12.8% in glucose readings at 10 and 40 mg/dL of maltose, respectively (Figure 4c).

Further analysis can be found in Table S8, along with the graphs showing all maltose concentrations (Figure S4) in the Supplemental Information.

#### 3.4.3. Acetaminophen

**Figure 5:**
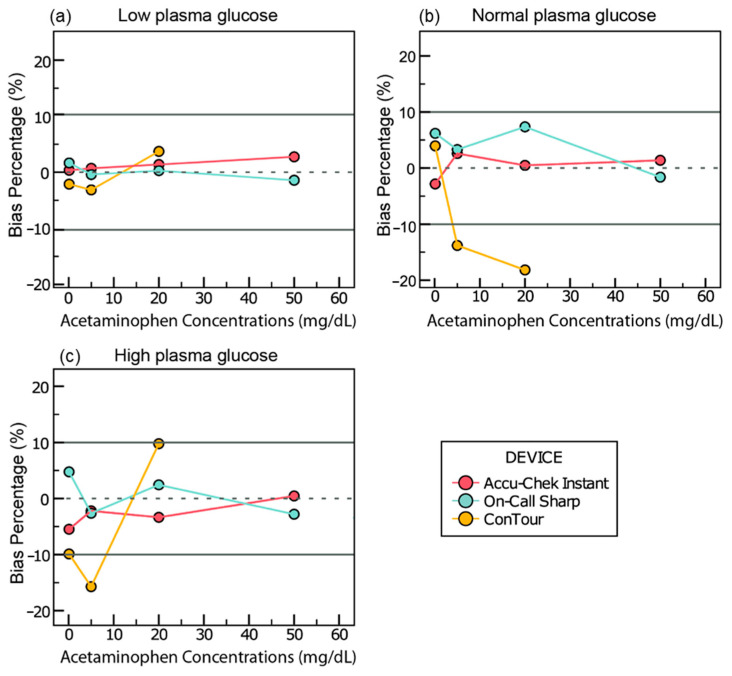
Bias and bias percentage of glucose readings across various acetaminophen concentrations. The X-axes of all graphs show acetaminophen levels. The Y-axes represent (**a**) the bias calculated as [glucometer reading in acetaminophen-spiked samples (mg/dL) − baseline reading (mg/dL)] for samples adjusted to 41.16 mg/dL of glucose and (**b**,**c**) the bias percentage calculated as [(glucometer with acetaminophen − baseline glucometer)/(baseline glucometer) × 100] for samples adjusted to 96.9 mg/dL and 413.26 mg/dL of glucose, respectively. Any missing values for ConTour are due to error messages.

In the samples adjusted to 41 mg/dL of glucose, all meters displayed a change between −10 and 10 mg/dL of glucose (Figure 5a). This change ranged from 0.3 to 3.3 mg/dL of glucose for Accu-Chek Instant, −2.4 to 1.6 mg/dL for On-Call Sharp, and −3.1 to 3.6 mg/dL for ConTour.

In the samples adjusted to 97 mg/dL of glucose, changes of −13.8% and −18.2% in glucose readings were calculated only with ConTour at 5 and 20 mg/dL of acetaminophen, respectively (Figure 5b).

In the samples adjusted to 413 mg/dL of glucose, ConTour showed a change in glucose readings of −15.7% at 5 mg/dL of acetaminophen and error messages at higher concentrations (Figure 5c). Throughout the three glycemic ranges, ConTour displayed E11 error messages when ≥ 50 mg/dL of acetaminophen was added.

Further analysis can be found in Table S9 along with graphs showing all acetaminophen concentrations (Figure S5) in the Supplemental Information.

## 4. Discussion

The purpose of this study was to determine the precision of three commonly used glucose meters in Saudi Arabia and the effects of vitamin C, maltose, and acetaminophen on the accuracy of the glucose readings of these devices. Our study revealed that Accu-Chek Instant, On-Call Sharp, and ConTour did not fully comply with the ISO standard throughout the precision experiment. Moreover, vitamin C yielded the greatest degree of interference among the different metabolites, with continuously positive biases beyond the ISO standard 15197 (2013) and values beyond the ISO standard ranging from 10.2 mg/dL of glucose to 116.2 mg/dL. Acetaminophen did not interfere with either the glucose readings of Accu-Chek Instant or those of On-Call Sharp beyond the ISO standard, but it did interfere with ConTour. Maltose did not interfere beyond the ISO standard with the glucose readings of all devices, with the exception of ConTour when the high blood pool was spiked with 10 and 40 mg/dL of maltose.

Similar to our findings, Salacinski and colleagues (2014) evaluated the precision of the Bayer ConTour glucose meter on capillary blood and reported that it showed high %CV values in the normal glucose concentrations (6.6%) and high glucose concentrations (5.3%) [20]. In addition, our results show that Accu-Chek Instant adhered to the ISO standard in the low, normal, and high glucose pools. However, Kermani and colleagues (2017) demonstrated that Accu-Chek Active, which is GDH-PQQ-based, lacks precision [21]. As in our study, Vanavanan and colleagues (2010) [22] and Tang and colleagues (2000) [14] reported acceptable %CV values across low, normal, and high glucose concentrations of 3.7%, 2.1%, and 3.6%, respectively, using heparinized whole-blood samples with Accu-Chek Advantage, which uses the GDH enzyme [14,22]. A similar pattern was observed in the low (3.09%), normal (1.69%), and high (0.89%) pools when Accu-Chek Aviva was used, which utilizes the GDH-PQQ enzyme [23].

In addition, vitamin C mostly positively deviated the glucose readings from the baseline beyond the ISO standards in all glucose plasma pools at the low to moderate interference concentrations in Accu-Chek and ConTour, respectively, and, to a great extent, in the normal and low pools. Several previous studies support our results, as they reported that vitamin C positively deviated the glucose readings of Accu-Chek systems, four of which reported glucose readings exceeding the ISO standards [19,21,24,25]. In contrast to our results in Accu-Chek, Lv and colleagues (2013) [26] reported that Roche Accu-Chek Inform showed a glucose change of less than 10% in all glucose whole-blood pools. Vanavanan and colleagues (2010) [22] suggested a possible explanation for the interference, stating that vitamin C could influence glucose readings because of the use of a disparate number of enzymes, technical methodology, or the structure of the test strips [22].

Maltose interferes with GDH-PQQ-based glucose meters [27] but not with FAD-dependent GDH glucose meters [28]. The three glucose meters used in this study utilize FAD-dependent GDH derived from the Aspergillus species, which has been shown to be maltose-insensitive [29]. Our study supports this statement except for ConTour, which appeared to falsely decrease the readings at low concentrations of maltose in the high plasma pool by percentages reaching approximately 5% beyond those of the ISO standards. Another study that used Barozen H Plus, a device that utilizes an FAD-dependent GDH enzyme, reported that maltose had an insignificant effect on the glucose readings in the low, normal, and high whole blood pools [10]. On the other hand, Roche Accu-Chek Inform showed positive bias by more than 10% when spiked with maltose [26]. However, GDH-PQQ-based glucose meters, as demonstrated by Vanavanan and colleagues (2010) [22], showed discrepant results that might be attributed to the origin of the enzyme or its formulation. Another study suggested poor precision as another possible reason [23].

In line with our results, which yielded an acceptable change in glucose readings with Accu-Chek Instant when spiked with acetaminophen, Lv and colleagues (2013) used Roche Accu-Chek Inform and yielded biases within the ISO standard [26]. However, one study that used Accu-Chek Advantage and Accu-Chek Advantage H showed positive biases that exceeded 10% [14].

A study conducted in Saudi Arabia on patients attending a primary healthcare center found that the prevalence of monitoring blood glucose in people diagnosed with diabetes is 95%. Moreover, intensive insulin therapy decreases the mortality rate in patients who are in an intensive care unit for more than five days. Also, it lowers the overall in-hospital mortality rate by 34% and several other comorbidities [30]. Hence, studies that provide data on the potential interferences and precisions of glucometers can potentially help physicians enhance patient care.

## 5. Conclusions

The precisions of the three commonly used glucometers in Saudi Arabia were measured through a series of experiments and statistical analyses, which led to the conclusion that only Accu-Chek Instant adhered to the ISO 15197 standards. As for interference, vitamin C yielded the greatest degree of change in glucose readings compared with maltose and acetaminophen, with continuously positive biases beyond the ISO standard, especially with ConTour.

## Data Availability

All data generated and analyzed during this study are included in this published article and its Supplemental Information files.

## Supplementary Materials

The following supporting information can be downloaded at: https://www.mdpi.com/article/10.3390/s25113561/s1. (References [31,32] are cited in the Supplementary Materials).
