# Supplementary material for: Determination of the Precision of Glucometers Used in Saudi Arabia
Source: Sensors (Basel). 2025 Jun 5;25(11):3561. doi: 10.3390/s25113561 (PMC12158352; doi:10.3390/s25113561)
Supplement: Supplementary file 1 [file sensors-25-03561-s001.zip › (Supplementary Tables) Determination of the Precision of Glucometers Used in Saudi Arabia.pdf]

Table S1. Dextrose concentrations that were used for the precision experiment. The choice of concentrations was based on the FDA guidelines, related to STAR★Methods.

Precision substance	# of aliquots	Concentrations (mg/dL)
Dextrose	5	20, 40, 60, 120, 400 (FDA, 2021)

Table S2. Spectrophotometer readings. Data collection of true glucose values allocated from the spectrophotometer, related to STAR★Methods.

Blood pool	True glucose values (mg/dL)
Low	41.16
Normal	96.9
High (before spiking with dextrose)	104
High (after spiking with dextrose)	413.26

Table S3. Summary statistics of the within-run glucose readings. Mean, standard deviation (SD), and coefficient of variation percentage (CV%) of glucose readings (mg/dL) of all blood pools for the three glucometer brands at different dextrose spikes used for the within-run precision experiment, related to Precision Experiment of the Results section.

Days	Blood pool	Device	Dextrose Concentrations (mg/dL)														
			20.00			40.00			60.00			120.00			400.00		
			Mean	SD	%CV	Mean	SD	%CV	Mean	SD	%CV	Mean	SD	%CV	Mean	SD	%CV
1	Normal	Accu-Chek Instant®	137.00	2.00	1.46	136.60	1.95	1.43	170.60	14.33	8.40	202.80	8.41	4.15	455.20	9.44	2.07
		On-Call Sharp®	134.80	7.33	5.44	161.80	3.77	2.33	179.00	10.56	5.90	219.80	9.01	4.10	516.00	17.87	3.46
		ConTour®	127.00	2.35	1.85	137.20	4.66	3.40	129.40	9.36	7.23	211.80	2.39	1.13	296.60	13.05	4.40
	Low	Accu-Chek Instant®	50.60	2.41	4.76	64.40	2.41	3.74	92.40	8.17	8.85	123.60	8.32	6.74	366.60	3.29	0.90
		On-Call Sharp®	47.40	4.93	10.40	61.60	4.22	6.85	88.00	7.11	8.08	121.40	10.88	8.96	393.60	7.89	2.01
		ConTour®	56.40	1.82	3.22	68.60	2.97	4.32	89.60	4.10	4.57	133.00	9.14	6.87	368.00	4.64	1.26
	High	Accu-Chek Instant®	492.60	36.76	7.46	518.00	13.60	2.63	518.80	23.19	4.47	553.80	24.25	4.38	.*	.*	.*
		On-Call Sharp®	480.40	11.06	2.30	502.40	22.24	4.43	524.00	38.09	7.27	542.60	11.91	2.19	.*	.*	.*
		ConTour®	443.00	25.50	5.76	495.00	16.26	3.29	496.60	61.31	12.35	476.60	24.13	5.06	.*	.*	.*
2	Normal	Accu-Chek Instant®	133.60	3.05	2.28	148.80	3.63	2.44	152.20	0.84	0.55	190.80	1.30	0.68	441.20	10.33	2.34
		On-Call Sharp®	131.40	5.94	4.52	153.20	5.22	3.40	159.80	2.77	1.74	216.80	5.17	2.38	440.00	14.23	3.23
		ConTour®	123.60	1.67	1.35	149.00	3.54	2.37	148.00	6.16	4.17	195.20	2.86	1.47	399.40	10.26	2.57
	Low	Accu-Chek Instant®	51.40	0.89	1.74	61.80	0.84	1.35	73.40	0.89	1.22	108.40	1.52	1.40	354.20	10.47	2.96
		On-Call Sharp®	50.00	2.00	4.00	57.80	1.48	2.57	78.60	4.22	5.37	122.40	6.50	5.31	380.60	9.29	2.44
		ConTour®	58.60	2.30	3.93	69.60	1.67	2.40	77.60	2.41	3.10	120.00	5.61	4.68	263.20	82.65	31.40
	High	Accu-Chek Instant®	488.00	13.11	2.69	516.00	22.95	4.45	524.40	23.56	4.49	562.40	10.33	1.84	.*	.*	.*
		On-Call Sharp®	503.20	17.63	3.50	525.60	31.60	6.01	549.20	18.16	3.31	454.40	25.43	5.60	.*	.*	.*
		ConTour®	479.80	16.42	3.42	480.80	42.45	8.83	515.20	46.84	9.09	338.40	32.19	9.51	.*	.*	.*
3	Normal	Accu-Chek Instant®	127.60	2.07	1.63	137.80	3.49	2.53	152.40	4.34	2.85	187.80	9.09	4.84	443.40	25.99	5.86
		On-Call Sharp®	132.40	1.14	0.86	151.00	15.44	10.23	149.40	3.51	2.35	205.80	12.48	6.06	462.40	13.03	2.82
		ConTour®	123.00	4.64	3.77	139.20	3.03	2.18	148.20	3.83	2.59	191.20	6.87	3.59	421.40	12.54	2.98

Days	Blood pool	Device	Dextrose Concentrations (mg/dL)														
			20.00			40.00			60.00			120.00			400.00		
			Mean	SD	%CV	Mean	SD	%CV	Mean	SD	%CV	Mean	SD	%CV	Mean	SD	%CV
3	Low	Accu-Chek Instant®	42.40	1.14	2.69	56.20	1.10	1.95	78.80	1.64	2.09	112.00	2.55	2.28	322.20	14.25	4.42
		On-Call Sharp®	35.80	0.84	2.34	51.00	1.41	2.77	78.80	2.68	3.41	121.40	2.70	2.23	329.20	9.52	2.89
		ConTour®	46.00	1.00	2.17	61.80	3.03	4.91	83.20	3.03	3.65	119.00	4.30	3.61	333.80	8.98	2.69
	High	Accu-Chek Instant®	452.80	6.53	1.44	453.80	6.22	1.37	516.20	6.53	1.27	520.00	16.09	3.09	.*	.*	.*
		On-Call Sharp®	486.80	3.56	0.73	489.40	11.93	2.44	511.80	15.47	3.02	569.80	11.41	2.00	.*	.*	.*
		ConTour®	476.20	14.97	3.14	479.80	15.35	3.20	476.40	10.21	2.14	108.40	.*	.*	.*	.*	.*

* Missing values due to error messages by glucometer.

Table S4. Summary statistics of the between-run glucose readings. Mean, standard deviation (SD), and coefficient of variation percentage (CV%) of glucose readings (mg/dL) of all blood pools for the three glucometer brands at different dextrose spikes used for the between-run precision experiment, related to Precision Experiment of the Results section.

Blood pool	Device	Dextrose Concentrations (mg/dL)														
		20.00			40.00			60.00			120.00			400.00		
		Mean	SD	%CV	Mean	SD	%CV	Mean	SD	%CV	Mean	SD	%CV	Mean	SD	%CV
Normal	Accu-Chek Instant®	132.73	4.61	3.47	141.07	6.37	4.52	158.40	12.00	7.58	193.80	9.45	4.88	446.60	17.01	3.81
	On-Call Sharp®	132.87	5.29	3.98	155.33	10.16	6.54	162.73	14.09	8.66	214.13	10.68	4.99	472.80	35.88	7.59
	ConTour®	124.53	3.44	2.76	141.80	6.39	4.51	152.00	8.80	5.79	199.40	10.13	5.08	429.77	39.64	9.22
Low	Accu-Chek Instant®	48.13	4.47	9.29	60.80	3.84	6.32	81.53	9.41	11.54	114.67	8.21	7.16	347.67	21.62	6.22
	On-Call Sharp®	44.40	7.01	15.78	56.80	5.19	9.13	81.80	6.49	7.94	121.73	6.94	5.70	367.80	29.95	8.14
	ConTour®	53.67	5.92	11.04	66.67	4.34	6.51	83.47	5.90	7.07	124.00	9.04	7.29	371.15	53.83	14.50
High	Accu-Chek Instant®	477.80	28.04	5.87	495.93	34.15	6.89	519.80	18.36	3.53	545.40	25.13	4.61	.*	.*	.*
	On-Call Sharp®	490.13	15.04	3.07	505.80	26.60	5.26	528.33	28.93	5.48	559.57	20.14	3.60	.*	.*	.*
	ConTour®	466.33	24.92	5.34	485.20	26.63	5.49	496.07	44.72	9.01	513.00	49.59	9.67	.*	.*	.*

* Missing values due to error messages by glucometer.

Table S5. Substance concentrations that were used in the interference experiment. The choice of concentrations of each substance was based on previous studies, related to STAR★Methods.

Interferences	# of aliquots	Concentrations (mg/dL)
Ascorbic acid (vitamin C)	6	5, 10, 25, 50, 100, 200 (Katzman et al., 2021)
Acetaminophen	6	0.1, 5, 20, 50, 80, 100 (Chenoweth et al., 2020)
Maltose	6	10, 40, 200, 480, 600, 800 (Cho et al., 2016)

Table S6. Descriptive statistics of samples spiked with vitamin C. The mean, standard deviation (SD), bias, bias percentage, and confidence intervals of glucose readings of plasma samples spiked with vitamin C, related to Interference Experiment of Results.

Blood Pool	Device	Concentration of Substance in Plasma (mg/dL)	Mean	SD	Bias (mg/dL)	Bias %	95% CI	
							LL	UL
Low	Accu-Chek Instant	5	33.67	0.58	2.33	7.45	4.65	13.96
		10	35.33	1.53	4	12.77	2.4	27.04
		25	49.67	1.53	18.33	58.51	48.94	73.58
		50	.*	.*	.*	.*	90.88	162.36
		100	.*	.*	.*	.*	.*	.*
		200	.*	.*	.*	.*	.*	.*
	On-Call Sharp	5	33	1.73	6.93	26.6	3.23	34.18
		10	39	2.65	12.93	49.62	16.65	63.93
		25	48.67	1.15	22.6	86.7	64.74	85.38
		50	63	4	36.93	141.69	49.7	58.14
		100	114	11.79	87.93	337.34	204.72	415.42
		200	140.67	4.93	114.6	439.64	361.92	450.07
	ConTour	5	38.33	0.58	0.6	1.59	8.53	16.96
		10	40	1	2.27	6.01	10.34	24.95
		25	38.33	1.53	22.6	86.7	1.58	23.91
		50	52.33	0.58	14.6	38.69	-6.54	-3.9
		100	.*	.*	.*	.*	.*	.*
		200	.*	.*	.*	.*	.*	.*
Normal	Accu-Chek Instant	5	111.33	4.04	0.53	0.48	-8.58	9.54
		10	111.67	0.58	0.87	3.79	-2.94	10.52
		25	115	3	4.2	3.79	-2.94	10.52
		50	135	4	24.2	21.84	12.87	30.81
		100	.*	.*	.*	.*	.*	.*
		200	.*	.*	.*	.*	.*	.*
	On-Call Sharp	5	120.67	2.31	5.47	4.75	-0.23	9.73

Blood Pool	Device	Concentration of Substance in Plasma (mg/dL)	Mean	SD	Bias (mg/dL)	Bias %	95% CI	
							LL	UL
		10	120.67	2.52	5.47	4.75	-0.68	10.17
Normal	On-Call Sharp	25	128	4.36	12.8	11.11	1.71	20.51
		50	134.33	1.53	19.13	16.61	13.31	19.9
		100	174.33	4.51	59.13	51.33	41.61	61.05
		200	189.33	17.79	74.13	64.35	26	102.7
	ConTour	5	113	3	-1.47	-1.28	-7.79	5.23
		10	105	4.58	-9.47	-8.27	-10.52	10.52
		25	100.33	0.58	-14.13	-12.35	-13.6	-11.09
		50	120.33	4.51	5.87	5.13	-4.66	14.91
		100	.*	.*	.*	.*	.*	.*
		200	.*	.*	.*	.*	.*	.*
High	Accu-Chek Instant	5	368	20.07	-20.6	-5.48	-7.47	-3.5
		10	371.33	10.02	-8.27	-2.2	-8.99	4.59
		25	375.67	2.08	-12.6	-3.35	-7.04	0.33
		50	356	2	1.73	0.46	-3.3	4.22
		100	421.33	8.5	-7.6	-2.02	-13.95	9.9
		200	.*	.*	-15.6	-4.15	-6.8	-1.51
	On-Call Sharp	5	371.67	17.21	-16.2	-4.18	-15.2	6.85
		10	368	2	-19.87	-5.12	-6.4	-3.84
		25	393.33	6.81	5.47	1.41	-2.95	5.77
		50	401	19.52	13.13	3.39	-9.12	15.89
		100	408.67	1.53	20.8	5.36	4.38	6.34
		200	410	16.37	22.13	5.71	-4.78	16.19
	ConTour	5	370	15.13	-23.53	-5.98	-15.53	3.57
		10	419	6.56	25.47	6.47	2.33	10.61
		25	437	12.12	43.47	11.05	3.39	18.7
		50	441	16.64	47.47	12.06	1.56	22.57
		100	.*	.*	.*	.*	.*	.*
		200	.*	.*	.*	.*	.*	.*

* Missing values due to error messages by glucometer.

Table S7. Descriptive statistics of samples spiked with maltose. The mean, standard deviation (SD), bias, bias percentage, and confidence intervals of glucose readings of plasma samples spiked with maltose, related to Interference Experiment of Results.

Blood Pool	Device	Concentration of Substance in Plasma (mg/dL)	Mean	SD	Bias (mg/dL)	Bias %	95% CI	
							LL	UL
Low	Accu-Chek Instant	10	31.67	0.58	0.33	1.06	-3.51	5.64
		40	32	1	0.67	2.13	-5.8	10.06
		200	32.67	0.58	1.33	4.26	-0.32	8.83
		480	34	1	2.67	8.51	0.58	16.44
		600	33.67	1.15	2.33	7.44	-1.71	16.6
		800	34.67	0.58	3.33	10.63	6.06	15.22
	On-Call Sharp	10	28	1	1.93	7.42	-2.11	16.95
		40	27.67	2.52	1.6	6.14	-17.85	30.12
		200	28.33	1.15	2.27	8.7	-2.31	19.7
		480	30	1.73	3.93	15.09	-1.42	31.6
		600	31.67	3.79	5.6	21.48	-14.6	57.56
		800	33	1	6.93	26.6	17.07	36.13
	ConTour	10	36	1	-1.73	-4.59	-11.18	1.99
		40	35.67	0.58	-2.07	-5.48	-9.28	-1.68
		200	35.67	0.58	-2.07	-5.48	-9.28	-1.68
		480	38.67	3.06	0.93	2.47	-17.64	22.59
		600	37.67	0.58	-0.07	-0.18	-3.98	3.62
		800	37.67	0.58	-0.07	-0.18	-3.98	3.62
Normal	Accu-Chek Instant	10	109.33	2.08	-1.47	-1.32	-5.99	3.34
		40	108.67	4.04	-2.13	-1.93	-10.99	7.14
		200	112	1	1.2	1.08	-1.16	3.33
		480	112.67	1.53	1.87	1.68	-1.74	5.11
		600	110.33	5.03	-0.47	-0.42	-11.71	10.86
		800	114	1.73	3.2	2.89	-1	6.77
	On-Call Sharp	10	111.67	3.21	-3.53	-3.07	-10	3.86
		40	118.67	5.77	3.47	3.01	-9.44	15.46
		200	110.67	4.62	-4.53	-3.94	-13.9	6.02
		480	114.33	1.15	-0.87	-0.75	-3.24	1.74
		600	115.33	2.08	0.13	0.12	-4.37	4.6
		800	113.33	1.53	-1.87	-1.62	-4.91	1.67

Blood Pool	Device	Concentration of Substance in Plasma (mg/dL)	Mean	SD	Bias (mg/dL)	Bias %	95% CI	
							LL	UL
	ConTour	10	107.67	1.15	-0.05	-0.04	-0.06	-0.02
Normal	ConTour	40	110.67	5.69	-0.02	-0.02	-0.13	0.09
		200	108	1	-0.05	-0.04	-0.06	-0.02
		480	111.67	4.62	-0.01	-0.01	-0.1	0.08
		600	111	6.08	-0.02	-0.02	-0.13	0.1
		800	115.33	1.53	0.02	0.02	-0.01	0.05
High	Accu-Chek Instant	10	355.67	3.06	-19.93	-5.31	-7.33	-3.29
		40	360	7.94	-15.6	-4.15	-9.4	1.1
		200	362.33	12.5	-13.27	-3.53	-11.8	4.74
		480	356.67	4.51	-18.93	-5.04	-8.02	-2.06
		600	362.67	0.58	-12.93	-3.44	-3.83	-3.06
		800	365	6.25	-10.6	-2.82	-6.95	1.31
	On-Call Sharp	10	362	6.93	-25.87	-6.67	-11.11	-2.23
		40	383	0	-4.87	-1.25	-1.25	-1.25
		200	372.33	5.03	-15.53	-4	-7.23	-0.78
		480	378.67	12.74	-9.2	-2.37	-10.53	5.79
		600	380.67	9.45	-7.2	-1.86	-7.91	4.2
		800	376.67	20.84	-11.2	-2.89	-16.24	10.46
	ConTour	10	335	15.72	-58.53	-14.87	-24.79	-4.95
		40	343	19.52	-50.53	-12.84	-25.16	-0.52
		200	366.33	16.62	-27.2	-6.91	-17.41	3.58
		480	382	29.82	-11.53	-2.93	-21.75	15.89
		600	375	3	-18.53	-4.71	-6.6	-2.82
		800	373.33	10.69	-20.2	-5.13	-11.88	1.62

Table S8. Descriptive statistics of samples spiked with acetaminophen. The mean, standard deviation (SD), bias, bias percentage, and confidence intervals of glucose readings of plasma samples spiked with acetaminophen, related to Interference Experiment of Results.

Blood Pool	Device	Concentration of Substance in Plasma (mg/dL)	Mean	SD	Bias (mg/dL)	Bias %	95% CI	
							LL	UL
Low	Accu-Chek Instant	0.1	31	1	0.33	1.06	-8.99	6.86
		5	32	1	0.67	2.13	-5.8	10.06
		20	31.33	0.58	1.33	4.26	-4.58	4.58

Blood Pool	Device	Concentration of Substance in Plasma (mg/dL)	Mean	SD	Bias (mg/dL)	Bias %	95% CI	
							LL	UL
		50	32.33	0.58	2.67	8.51	-1.39	7.77
		80	31.67	1.53	2.33	7.45	-11.05	13.17
Low	Accu-Chek Instant	100	31.67	1.53	3.33	10.64	-11.05	13.17
	On-Call Sharp	0.1	27.67	2.08	1.6	6.14	-13.7	25.98
		5	25.67	1.53	-0.4	-1.53	-16.09	13.02
		20	26.33	1.53	0.27	1.02	-13.53	15.58
		50	24.67	1.53	-1.4	-5.37	-19.93	9.19
		80	24.33	0.58	-1.73	-6.65	-12.15	-1.15
		100	26.33	4.93	-2.4	-9.21	-14.71	-3.71
	ConTour	0.1	35.67	1.15	-2.07	-5.48	-13.08	2.12
		5	34.67	1.53	-3.07	-8.13	-18.18	1.93
		20	41.33	1.53	3.6	9.54	-0.52	19.6
		50	.*	.*	.*	.*	.*	.*
		80	.*	.*	.*	.*	.*	.*
		100	24	.*	.*	.*	.*	.*
Normal	Accu-Chek Instant	0.1	107.67	0.58	-3.13	-2.83	-4.12	-1.53
		5	113.67	3.21	2.87	2.59	-4.62	9.79
		20	111.33	1.53	0.53	0.48	-2.94	3.91
		50	112.33	1.53	1.53	1.38	-2.04	4.81
		80	111.33	2.08	0.53	0.48	-4.19	5.15
		100	108.67	3.21	-2.13	-1.93	-9.13	5.28
	On-Call Sharp	0.1	122.33	6.51	7.13	6.19	-7.84	20.22
		5	119	4.36	3.8	3.3	-6.1	12.7
		20	123.67	2.89	8.47	7.35	1.12	13.57
		50	113.33	3.79	-1.87	-1.62	-9.78	6.54
		80	111	3.61	-4.2	-3.65	-11.42	4.13
		100	111.33	3.79	-3.87	-3.36	-11.52	4.81
	ConTour	0.1	119	3.61	4.53	3.96	-3.86	11.79
		5	98.67	0.58	-15.8	-13.8	-15.06	-12.55
		20	93.67	1.53	-20.8	-18.17	-21.49	-14.86
		50	.*	.*	.*	.*	.*	.*
		80	.*	.*	.*	.*	.*	.*

Blood Pool	Device	Concentration of Substance in Plasma (mg/dL)	Mean	SD	Bias (mg/dL)	Bias %	95% CI	
							LL	UL
		100	.*	.*	.*	.*	.*	.*
High	Accu-Chek Instant	0.1	355	3	-20.6	-5.48	-7.47	-3.5
		5	367.33	10.26	-8.27	-2.2	-8.99	4.59
		20	363	5.57	-12.6	-3.35	-7.04	0.33
		50	377.33	5.69	1.73	0.46	-3.3	4.22
		80	368	18.03	-7.6	-2.02	-13.95	9.9
		100	360	4	-15.6	-4.15	-6.8	-1.51
	On-Call Sharp	0.1	406.33	13.05	18.47	4.76	-3.6	13.12
		5	377.67	14.98	-10.2	-2.63	-12.22	6.93
		20	397.33	14.57	9.47	2.44	-6.89	11.77
		50	377	8	-10.87	-2.8	-7.93	2.32
		80	371.33	12.58	-16.53	-4.26	-12.32	3.8
		100	363.33	15.28	-24.53	-6.33	-7.51	3.46
	ConTour	0.1	354.67	22.81	-38.87	-9.88	-24.28	4.52
		5	331.67	3.06	-61.87	-15.72	-17.65	-13.79
		20	432	16.09	38.47	9.77	-0.38	19.93
		50	.*	.*	.*	.*	.*	.*
		80	.*	.*	.*	.*	.*	.*
		100	.*	.*	.*	.*	.*	.*

* Missing values due to error messages by glucometer.

Table S9. Technical details for each glucometer brand and its test strips, related to STAR★Methods.

Monitor name	Generation	Manufacturer	Detectable range (mg/dL)	Device code	Batch number (LOT)*	Test strip enzyme	Number of tests strips per bottle	Expiry date (test strip)
Accu-Chek Instant®	2nd	Roche	10-600	No coding	301640	Glucose dehydrogenase FAD	50	05/2023
On-Call Sharp®	2nd	OnCall	10-600	OGS-121	1090143	Glucose dehydrogenase FAD	50	10/2023
ConTour®	2nd	Ascensia	10-600	No coding	DP1FJ3801E	Glucose dehydrogenase FAD	50	06/2023

References

Cho, J., Ahn, S., Yim, J., Cheon, Y., Jeong, S.H., Lee, S.-G., and Kim, J.-H. (2016). Influence of vitamin C and maltose on the accuracy of three models of glucose meters. Ann. Lab. Med. 36(3), 271–274.

Chenoweth, J., Dang, T., Gao, G., Tran, N. (2020). Acetaminophen interference with Nova StatStrip® Glucose Meter: case report with bench top confirmation. Clinical Toxicology. 58(11), 1067-1070.

Katzman, B., Kelley, B., Deobald, G., Myhre, N., Agger, S. and Karon, B. (2020). Unintended Consequence of High-Dose Vitamin C Therapy for an Oncology Patient: Evaluation of Ascorbic Acid Interference With Three Hospital-Use Glucose Meters. J. Diabetes. Sci. Technol. 15(4), 897-900.

"Self-Monitoring Blood Glucose Test Systems for Over-the-Counter Use", (2020), U.S. Food and Drug Administration, https://www.fda.gov/regulatory-information/search-fda-guidance-documents/self-monitoring-blood-glucose-test-systems-over-counter-use.
