# Supplementary material for: Determination of the Precision of Glucometers Used in Saudi Arabia
Source: Sensors (Basel). 2025 Jun 5;25(11):3561. doi: 10.3390/s25113561 (PMC12158352; doi:10.3390/s25113561)
Supplement: Supplementary file 1 [file sensors-25-03561-s001.zip › Supplementary Figure S1.pdf]

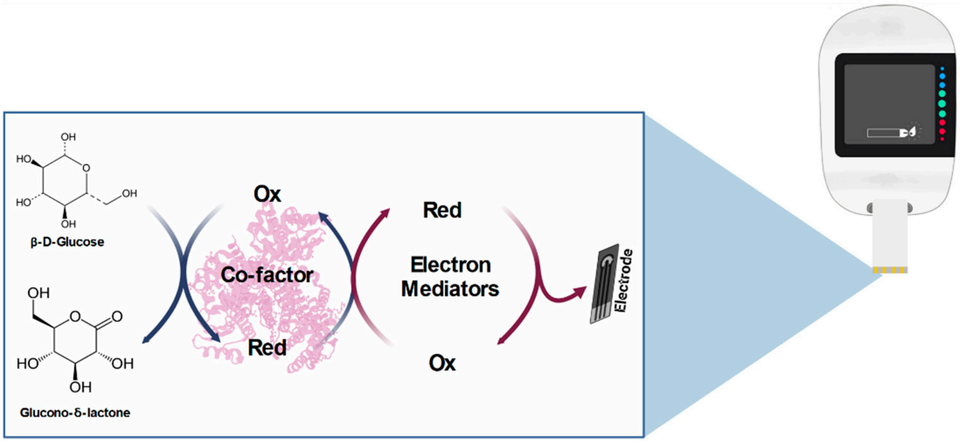

Supplementary Figure S1. Redox reaction at the level of the test strip. The reaction is composed of a reductive (blue) and an oxidative half (red). In the reductive half, β-D-glucose gets oxidized to glucono-δ-lactone by an enzyme, while its cofactor gets reduced. In the oxidative half, the cofactor reduces an electron mediator, which in turn transfers electrons to the electrode to generate a current that will be turned into a reading displayed on the LCD panel. Different biosensors differ mainly in the oxidative half. For the first-generation biosensors, an immobilized glucose oxidase (GOx) is utilized, and its reduced cofactor reduces oxygen to hydrogen peroxide. In second-generation biosensors, the cofactor reduces an electron mediator, which in turn transfers electrons to the electrode. For the third-generation, the glucometer utilizes a direct electron transfer mechanism where electrons are transferred immediately from glucose to the electrode.
