# Supplementary material for: Determination of the Precision of Glucometers Used in Saudi Arabia
Source: Sensors (Basel). 2025 Jun 5;25(11):3561. doi: 10.3390/s25113561 (PMC12158352; doi:10.3390/s25113561)
Supplement: Supplementary file 1 [file sensors-25-03561-s001.zip › Supplementary Figure S2.pdf]

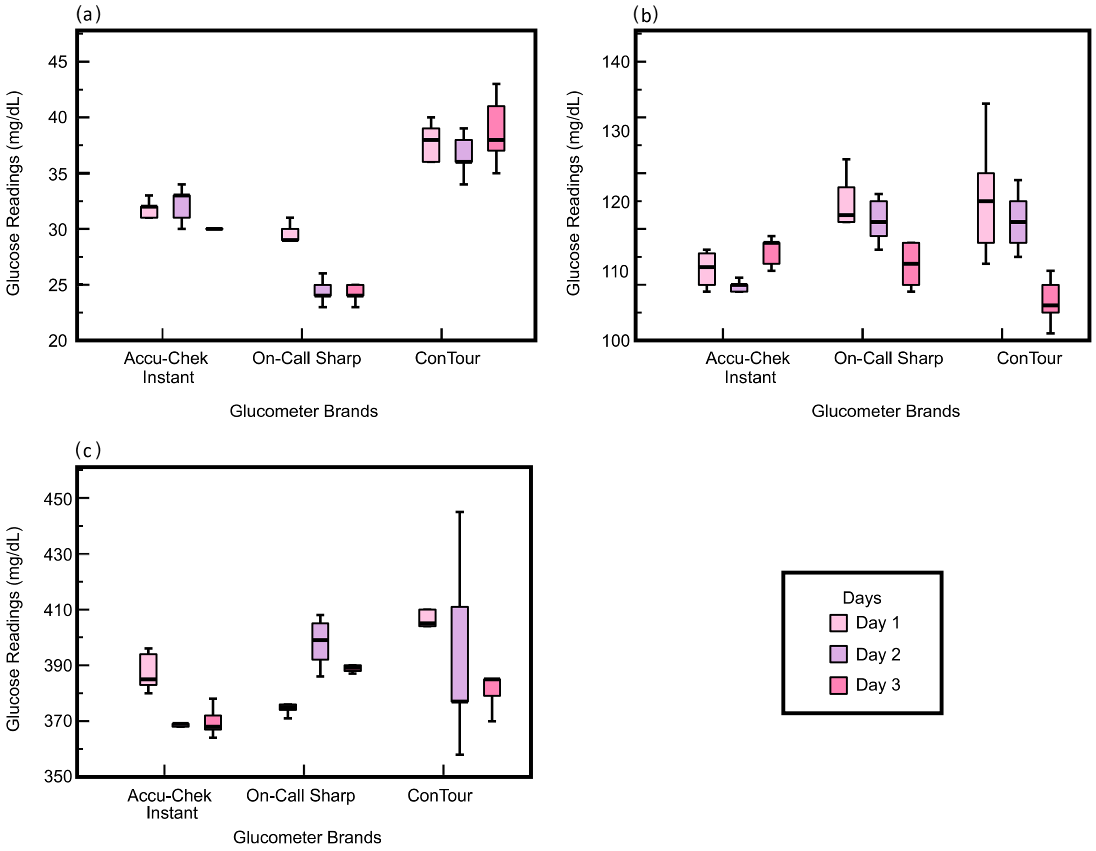

Supplementary Figure S2. Boxplot for baseline glucose readings. (a) Baseline readings across 3 days for the low blood pool. (b) Baseline readings across 3 days for the normal blood pool. (c) Baseline readings across 3 days for the high blood pool.
