# Supplementary material for: Determination of the Precision of Glucometers Used in Saudi Arabia
Source: Sensors (Basel). 2025 Jun 5;25(11):3561. doi: 10.3390/s25113561 (PMC12158352; doi:10.3390/s25113561)
Supplement: Supplementary file 1 [file sensors-25-03561-s001.zip › Supplementary Figure S5.pdf]

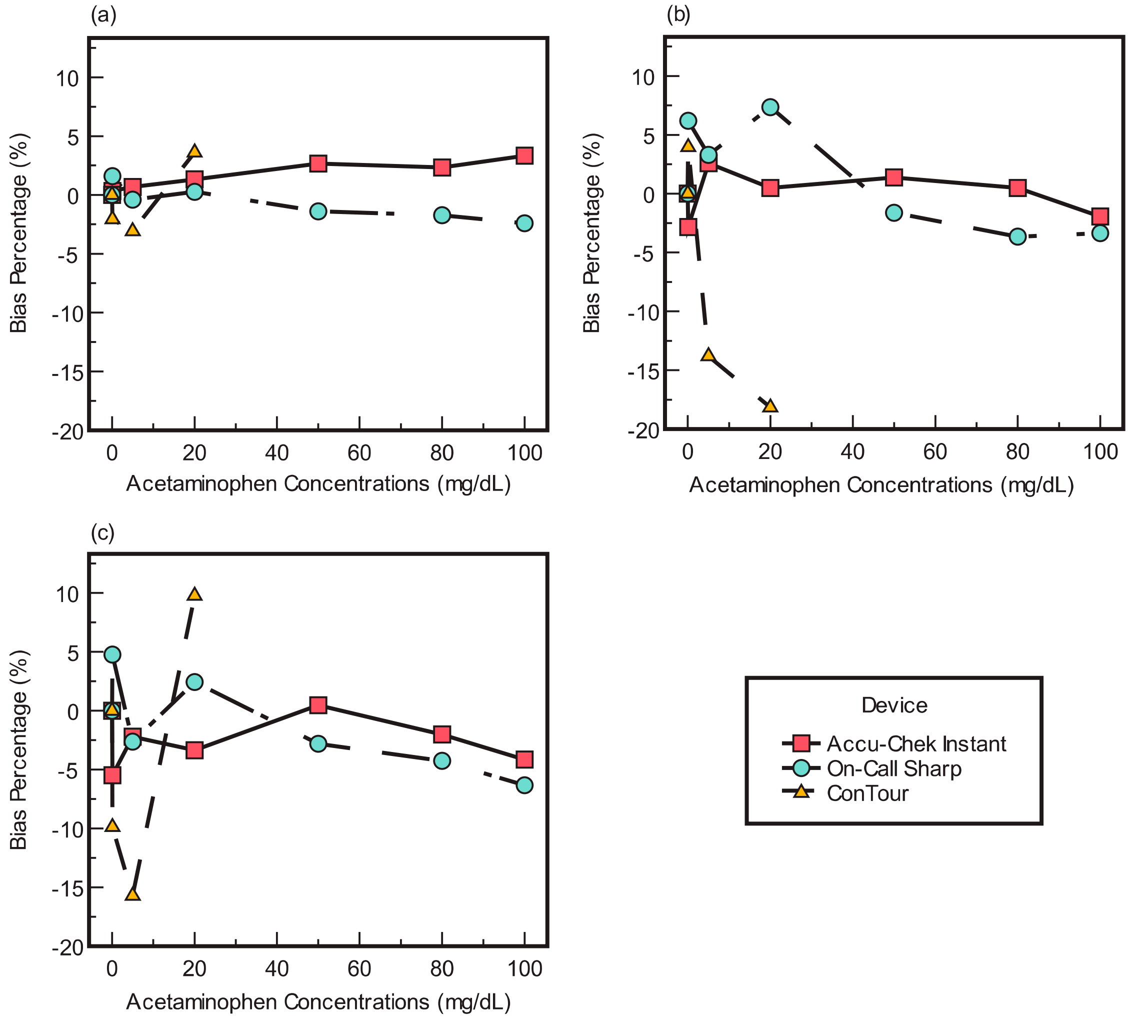

Figure S5. Bias and bias percentage of glucose readings across various acetaminophen concentrations, related to Interference Experiment of Results. The X-axis on all graphs shows the levels of acetaminophen. The Y-axis represents:

(a) the bias for samples spiked with acetaminophen, calculated as the glucometer reading with acetaminophen (in mg/dL) minus the baseline glucometer reading (in mg/dL), for samples adjusted to 41.16 mg/dL of glucose.

(b) and (c) display the bias percentage, calculated as [(glucometer reading with acetaminophen - baseline glucometer reading) / baseline glucometer reading] × 100, for samples adjusted to 96.9 mg/dL and 413.26 mg/dL of glucose, respectively. Any missing values for ConTour are due to error messages.
